# E-procurement in support of universal health coverage

**DOI:** 10.2471/BLT.15.020315

**Published:** 2015-03-01

## Abstract

Kenya is gearing up for digital bidding on essential medicines’ contracts, part of a wave of African countries looking at procurement to improve transparency, bring down costs and support universal health coverage. Gary Humphreys reports.

John Kabuchi, procurement manager for the Kenya Medical Supplies Authority, can feel it coming. “We are currently gearing up for full e-procurement functionality, including electronic bidding, and I am hopeful that supporting legislation will be passed before next June,” he says.

Kenya introduced social health insurance in recent years and hopes to make the most of new technologies and approaches, such as e-procurement, to support efforts to make essential health care more widely available. 

Essential medicines account for a large part of the health budget and so countries need to get the best deals when they purchase these in bulk. 

The Kenya Medical Supplies Authority is an autonomous agency that is responsible for keeping the country’s public health facilities stocked with essential medicines, and already makes limited use of the internet for the tender process. 

But, says Kabuchi, for the time-being and, in accordance with current legislation, bids still have to be submitted on paper.

As arcane as that might sound, paper-based bidding for government contracts is still commonplace in most parts of the world.

“Government medicines’ contracts can be extremely lucrative and there is plenty of scope for collusion and fraud,” explains Lisa Hedman, a procurement and supply chains expert at the World Health Organization (WHO).

“So what you don’t want is a system influenced by informal deals going on over the telephone and the like.” 

Paper bidding helps to keep the procurement process above board. In many countries, Kenya included, suppliers bidding on contracts are obliged to gather in a room, where their bids are opened for all to see.

Well intentioned as such efforts may be, they have a limited impact on illegal activities such as bid-rigging.

Bid rigging takes a variety of forms, including “market allocation”, when cartel members divide the market and decide in advance which firms should submit the winning bids and “bid rotation”, when conspiring firms bid, but agree to take turns at being the winner. 

“Bid rigging is a huge problem and no country is immune,” says Leonardo Noyola, a procurement consultant with the Organisation for Economic Co-operation and Development (OECD) in Mexico, and one of the authors of a recent OECD report on the subject.

“Bid rigging is a huge problem and no country is immune.”

Leonardo Noyola

“Up to one third of the money spent by governments on medicines can go into the pockets of cartels,” Noyola says. “Given that the health-care sector represents up to 6% of the GDP in OECD countries, losses can be considerable.”

Obliging people to open paper bids in a closed room is not going to change that. “In fact, this practice facilitates collusion,” Noyola says, “because sensitive information is disclosed – like the biddings offered by competitors – and it’s easier for cartel members to monitor the compliance with collusive agreements.”

“The great thing about e-procurement is that it increases competition by reducing the participation cost for bidders, maximizing their potential participation, and reducing communication among them,” he says.

Paper bids are also supposed to help establish a paper trail, giving buyers and sellers a means of keeping track of prices paid over extended periods. But here too, the reality falls short of the intention.

Paper bids are easily lost, misplaced or destroyed – something that is more likely to happen where corruption is a factor – making the paper trail difficult to follow.

Electronic record-keeping is one of the advantages of going digital.

Electronic procurement or e-procurement essentially means using some sort of digital, typically web-based platform to post tenders and receive bids.

Depending on how the system is set up, electronic bids are not just permanent but public – an important consideration where transparency and accountability are policy objectives. 

It is partly to address such issues that Kabuchi is keen to see the Kenya Medical Supplies Authority’s e-procurement capacity developed. “We expect turnaround time for the bidding process to be quicker, but it is clear that one of the main benefits will be increased transparency and trace-ability,” he says.

Going digital is not just about improving transparency.

Some argue that it allows procurement agencies to get better prices by throwing tenders open to more bidders – with so-called open-tender procurement, for example, where quotes can be invited from suppliers on a worldwide basis.

For WHO’s Hedman the real benefit of e-procurement is that it gives procurement agencies more choice and it is more transparent for potential bidders. Moreover, it can be used at national levels, but also expands the options for procurers operating further down in the supply chain.

“It’s not about getting more bids, it’s getting more relevant bids,” she says. “If I’m a decentralized procurement manager … in a district, I may not be interested in getting bids from a supplier whose warehouse is located on the other side of the country. I may be much more interested in getting bids from a local supplier with a manufacturing facility I can actually go and visit.”

Buyers may also prefer to do business with local suppliers who are more likely to have met domestic regulatory or registration requirements.

E-procurement can have a direct impact on price where a bidding mechanism, known as reverse auction, is used. In a reverse auction, suppliers submit initial bids on a contract, the lowest of which is posted publicly without naming the supplier.

In the second round, suppliers who are still interested bid below the posted low price and so it goes on until no more bids are submitted. Thus the lowest price established in the penultimate round wins the contract. To date, Latin American countries have been most active in developing reverse auction procurement, Ecuador being a prime example.

According to Francisco Páez, a former director at the National Institute of Public Purchases, the main public purchasing entity in Ecuador, reverse auctions were introduced following the election of President Rafael Correa, who took office in January 2007.

“That same year we started working on e-procurement and invested US$ 1 300 000 in information technology capacity,” says Páez. “For us it seemed logical to develop reverse auction from the start – it was the best way of establishing the real price.” Three years later the system was up and running.

“It was the best way of establishing the real price.”

Francisco Páez

The new government also took the opportunity to group together different buyers. The health sector in Ecuador is made up of four major institutions with responsibility for around 2800 public health establishments.

Roughly 1200 of them used to make purchases on their own. This had an impact on the prices they could achieve and adversely affected planning, efficiency and control.

“We brought them all under one umbrella, which allowed us not only to get better prices – with savings of around 40% – but also better quality, delivery times, logistics services and storage,” Páez says.

Other Latin American countries to have embraced e-procurement include Brazil, Chile, Colombia and Paraguay, and, most recently, Mexico, where the Mexican Institute of Social Security, the single largest purchaser of pharmaceutical products and other medical supplies in Latin America, has started to use reverse auctions and consolidate purchases for the procurement of medicines in the hope of achieving significant savings.

Where Latin American countries have led, African countries may now follow.

South Africa has been using e-procurement for some time, and, as already noted, Kenya is preparing to take the plunge. According to Kabuchi, representatives from several African countries, including Ethiopia, Malawi, Rwanda and Uganda have been in touch with him to discuss the challenges and possibilities, while Zambia too may be close to going digital.

According to Boniface Fundafunda, managing director of Zambia’s Medical Stores Limited, an autonomous government agency tasked with supplying high quality drugs and medical equipment at affordable prices, the Zambia Public Procurement Authority is in the process of considering submissions from stakeholders regarding the issue of e-procurement.

“There are indications that new legislation will follow,” Fundafunda says. “It seems likely that 2015 will be the year in which e-procurement shall be put into effect.”

**Figure:**
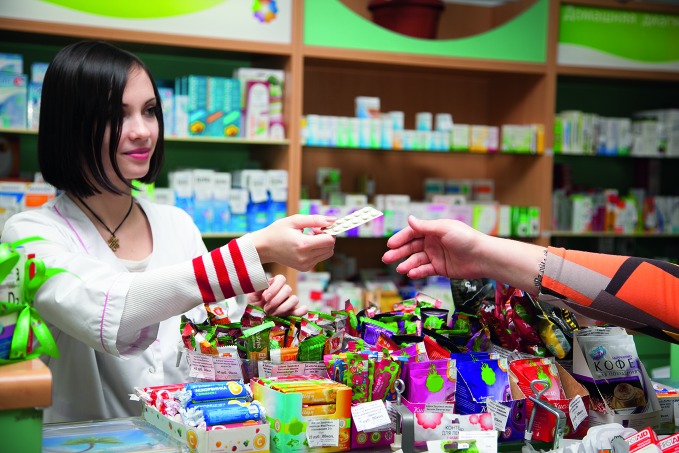
Medicines – such as those sold in this Russian pharmacy in Moscow – often take up a large part of government heath budgets.

WHO/Sergey Volkov

On the path to universal health coverage, all countries must strive to ensure that funds for health are spent wisely. “Governments are recognizing that incorporating information and communication technologies is a priority for health systems development,” says Joan Dzenowagis, an e-health expert at WHO. 

“Electronic systems and tools enable the efficient and accountable delivery of essential supplies – drugs, vaccines and equipment – through the management of procurement, supply and distribution chains. 

“But they don’t stand alone, and building the capacity and enabling environment around information systems remains a critical need,” Dzenowagis says.

Kenya is a case in point. According to a recent official statement, at best only 65% of the public health facilities’ needs are met with government resources, the balance being bridged with contributions from donors, and funds the health facilities can raise by charging user fees.

The government says that the problem is being addressed by increasing allocations to the health sector and that when the Abuja Declaration target – of 15% of total government budget going to the health sector – is met, “the funding gap for procurement of medical commodities will be eliminated”. The Kenya Medical Supplies Authority, and institutions like it, can help reduce that gap with e-procurement, but they can’t close it on their own.

